# A parallel solution to finding nodal neighbors in generic meshes

**DOI:** 10.1016/j.mex.2020.100954

**Published:** 2020-06-09

**Authors:** Pian Qi, Gang Mei, Nengxiong Xu, Hong Tian

**Affiliations:** a School of Engineering and Technology, China University of Geosciences (Beijing), 100083, Beijing, China; b Faculty of Engineering, China University of Geosciences (Wuhan), 430074, Wuhan, China

**Keywords:** Computational geometry, Mesh topology, Neighbors finding, Parallel Programming, GPU

## Abstract

In this paper we specifically present a parallel solution to finding the one-ring neighboring nodes and elements for each vertex in generic meshes. The finding of nodal neighbors is computationally straightforward but expensive for large meshes. To improve the efficiency, the parallelism is adopted by utilizing the modern Graphics Processing Unit (GPU). The presented parallel solution is heavily dependent on the parallel sorting, scan, and reduction. Our parallel solution is efficient and easy to implement, but requires the allocation of large device memory.

• Our parallel solution can generate the speedups of approximately 55 and 90 over the serial solution when finding the neighboring nodes and elements, respectively.
• It is easy to implement due to the reason it does not need to perform the mesh-coloring before finding neighbors
• There are no complex data structures, only integer arrays are needed, which makes our parallel solution very effective.

## Graphical abstract

**Figure:**
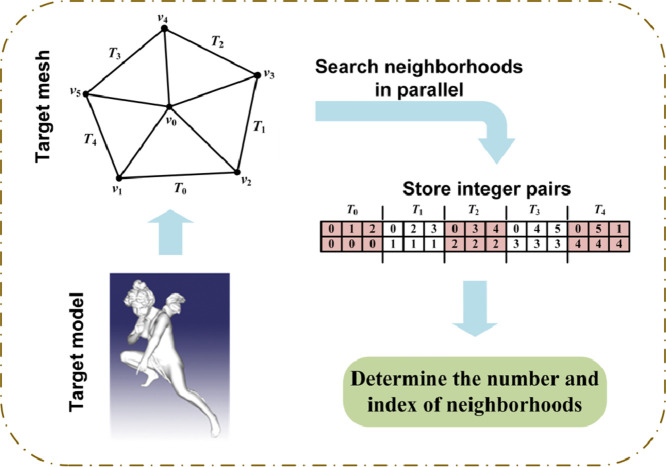

**Table:** Specifications Table

Subject Area:	Computer Science
More specific subject area:	Algorithm Design
Method name:	Parallel Neighbors Finding
Name and reference of original method:	N/A
Resource availability:	N/A

## Method details

In this section, we will describe the basic ideas and implementation details of our parallel solution to finding the one-ring neighboring nodes and elements for each vertex in generic meshes.

## Parallel solution to finding neighboring nodes for each vertex

**Fig. 1:**
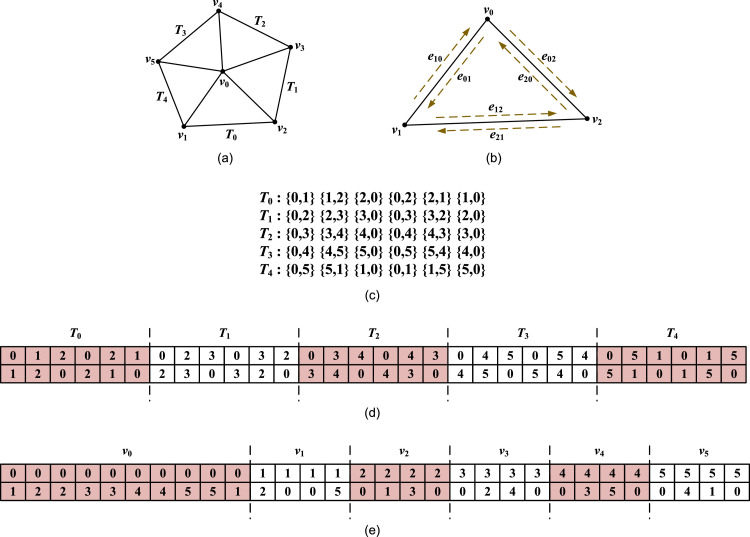
A simple illustration of concurrently finding neighboring nodes for each vertex in a mesh.

The basic ideas for concurrently finding neighboring nodes for each vertex are as follows. In any valid meshes, any pair of neighboring nodes is connected using an edge. An edge can be represented with two nodes (and further, the indices of two nodes). A mesh typically has plenty of edges (i.e., pairs of nodes). When gathering all edges of a mesh (see Fig. 1(a) and 1(b)), the edges can be stored in an array consisting of n pairs of integer values; see Fig. 1(c). The array of edges can be also considered as two arrays of integers (Fig. 1(d)). If adopting the first array of integers as the keys for sorting and the second array and the correspondingly attached values, the indices of all the neighboring nodes for the same vertex can be easily found by performing a parallel sorting; see Fig. 1(e).

## Parallel solution to finding neighboring elements for each vertex

**Fig. 2:**
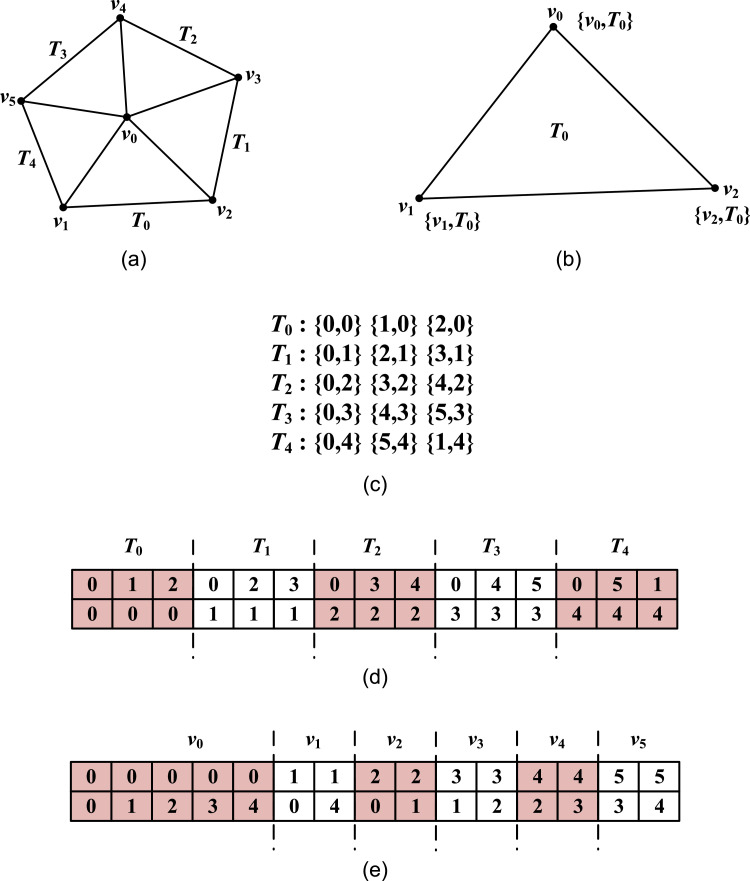
A simple illustration of concurrently finding neighboring elements for each vertex in a mesh.

The basic ideas for concurrently finding neighboring elements for each vertex are as follows. An element in a mesh is composed of several nodes (Fig. 2(a)). Each element is by nature the one-ring neighboring element of those nodes it contains. A pair of integer values can be used to simply demonstrate this relationship: the first integer is the index of one of the nodes in an element; and the second is the index of the element itself; see Fig. 2(b).

For an element, several such pairs of integers can be formed. And for an arbitrary mesh, a group of such pairs of integers can be obtained, and stored in two arrays of integers. Similar to the finding of neighboring nodes for each vertex, if adopting the first array of integers as the keys for sorting and the second array of integers as the correspondingly attached values, the indices of all the neighboring elements for the same vertex can be easily found by performing a parallel sorting; see Fig. 2.

## Implementation details

Our solution is applicable to arbitrary meshes. However, to demonstrate our solution, here we specifically present the implementation details of our solution when applied to the triangular surface mesh.

Our solution is implemented by strongly utilizing the parallel primitives provide by the library *Thrust* [1] such as the parallel sorting and reduction. More implementation details are listed as follows.

### The finding of neighboring nodes

The process of finding the neighboring nodes for each vertex is as follows.

First, we create pairs of integers according to the edges in each triangle. Noticeably, we can form three edges (pairs of integers) for a triangle when the three nodes in the triangle are organized in count-clockwise (CCW) order and another three pairs when nodes are organized in clockwise (CW); see Fig. 1(b). That is, a triangle can produce six pairs of integers. These edges/pairs can be obviously created in parallel. We specifically design a CUDA kernel to realize this.

After creating the pairs of integers that are stored in two arrays of integers, the second step is to sort those pairs according to the first array of integers. This procedure can be extremely fast performed by using the specific function thrust::sort_by_keys().

The third step is to determine: (1) the total number and (2) the detailed indices of the neighboring nodes for each vertex, which can be realized by using segmented scan and reduction. The ideas behind performing the segmented scan and reduction have been presented in our previous work [2].

To determine the number of neighbors, we first create a helper array containing the same value 1, and then perform a parallel segmented reduction by using the function thrust::reduce_by_keys(). To obtain the indices of the neighboring nodes, we also first create a helper array of sequenced integers, and then perform a parallel segmented scan by using thrust::unique_by_keys(). After performing the segmented reduction and scan, both the number and indices of neighbors can be found and then transferred into other target arrays for further mesh editing such as Boolean operations or mesh optimization. The relevant code is shown in Code 1.

### The finding of neighboring elements

The process of finding the neighboring elements is quite similar to that of finding the neighboring nodes. The first step is also to form the pairs of integers (i.e., two arrays of integers), then to sort according to the first array of integers, and third use the parallel segmented reduction and scan to further determine both the total number and the detailed indices of the neighboring elements.

However, there is a remarkable difference between the process of finding neighboring nodes and elements. When finding the neighboring elements, the first integer value of any pair is the index of a node in an element; and the second value of the pair of integers is the index of the element itself. In contrast, in the finding of neighboring nodes, both the first and the second integer value of any pair is the index of node.

## Method validation

### Results

Six groups of experimental tests are carried out to evaluate the performance of our parallel solution. These experimental tests of the parallel solution are performed on the desktop computer which features with the NVIDIA GeForce GT640 (GDDR5) graphics card and the programming model CUDA v7.0. The experiments of the corresponding serial solution are performed on Windows 7 SP1 with an Intel i5–3470 CPU (3.2 GHz and 4 Cores) and 8GB of RAM memory.

**Fig. 3:**
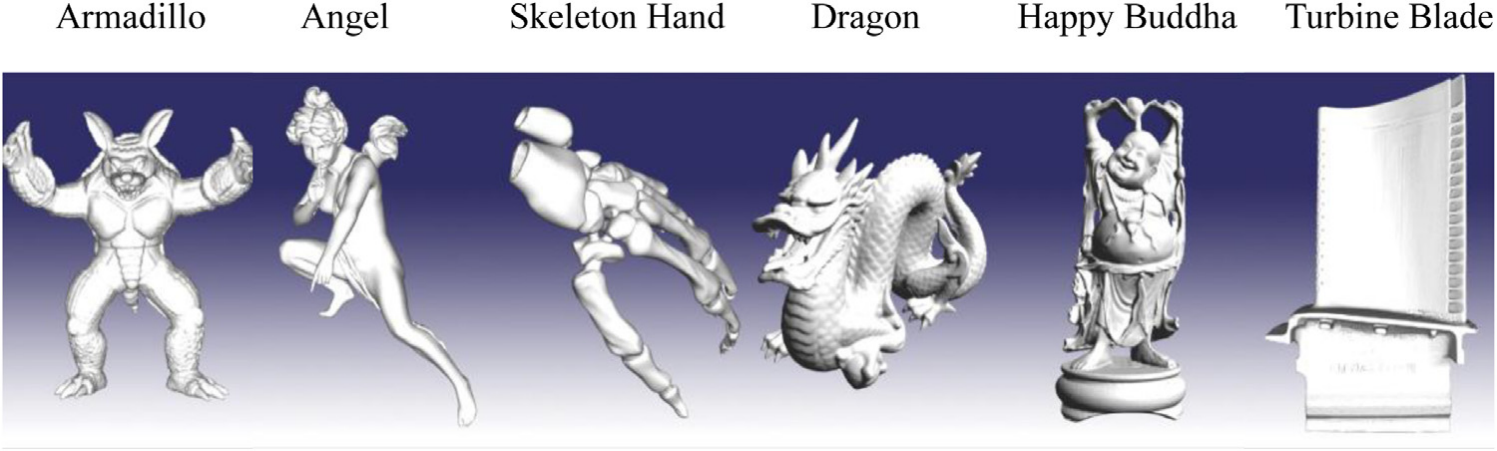
Six models for testing.

These six triangular surface mesh models employed for testing are directly obtained from the Stanford 3D Scanning Repository (http://www.graphics.stanford.edu/data/3Dscanrep/) and the GIT Large Geometry Models Archive (http://www.cc.gatech.edu/projects/large_models/); see Fig. 3.

The execution time of both our parallel solution and the serial solution for finding the neighboring nodes and elements are listed in Table 1. The experimental results indicate that: our parallel solution is approximately 55 and 90 times faster than the serial solution when finding the neighboring nodes and elements, respectively.

**Table 1:** Comparison of efficiency of our parallel solution and the serial solution (1 k = 1000).

Mesh Model	Num. of Node	Num. of Elem.	Serial (/ms)		Parallel (/ms)		Speedup	
			Find Node	Find Elem.	Find Node	Find Elem.	Find Node	Find Elem.
Armadillo	172 k	346 k	2527	1732	50.0	22.1	50.5	78.4
Angel	237 k	474 k	3635	2580	66.7	29.4	54.5	87.8
Hand	327 k	655 k	4806	3305	89.1	38.3	53.9	86.3
Dragon	437 k	871 k	6543	4488	112.7	48.5	58.1	92.5
Buddha	543 k	1088 k	8577	6072	138.5	60.6	61.9	100.2
Blade	882 k	1765 k	13,931	10,000	221.3	93.3	63.0	107.2

**Code 1:** Finding neighboring nodes in parallel.

## Discussion

### The advantages of our parallel solution

The first advantage of our parallel solution is that: it is easy to implement due to the reason it does not need to perform the mesh-coloring before finding neighbors. The mesh coloring technique is frequently used to deal with the race condition issue [3].

When finding nodal neighbors in parallel, there exists the race condition. The race condition issue appears when two different parallel threads may need to be written in the same memory position [3]. When looping over all the elements in a mesh to find the nodal neighbors, two neighboring nodes of the same vertex may be found concurrently within two parallel threads; and the indices of the two neighboring nodes may need to be written in the same memory position for storing. In this case, race condition arises.

Currently, the most commonly used method to address the above problem is to color the mesh first and then looping over those elements with the same color simultaneously to find neighbors [3,4,5,6,7]. This coloring-based method is very effective and efficient for large size of meshes, and is quite suitable to be applied in parallel pattern. The only minor shortcoming is that: it is needed to color the mesh into several groups of elements and thus needs additional computational cost.

In our parallel solution, we have redesigned the process of finding neighbors to avoid the use of mesh-coloring by strongly exploiting those efficient parallel primitives such as parallel sorting and scan. In addition, there are no complex data structures; and only arrays of integers are needed. Thus, our parallel solution is easy to implement in practice.

The second advantage of our parallel solution is the acceptable efficiency. The experimental results listed in Table 1 indicate that: our parallel solution can generate the speedups of approximately 55 and 90 over the serial solution when finding the neighboring nodes and elements, respectively.

This performance gains benefit from the parallelization performed on the GPU. By analyzing and reorganizing the process of finding nodal neighbors, we have transferred the entire process into several sub-procedures of parallel sorting, scan, and reduction, while these parallel primitives are extremely fast for the large size of data.

Another reason why our parallel solution is quite efficient is that: there are no complex data structures; and only arrays of integers are needed. Inherently, operations and computations for discrete arrays of integer values rather than arrays of structures are quite fast on the GPU. We specifically avoid using arrays of structures such as pairs, but chose to directly use the arrays of values. This leads additional performance gains.

### The shortcomings of our parallel solution

Although our parallel solution is efficient and easy to implement, it has an obvious shortcoming, i.e., it requires more device memory than that of the serial solution. This additionally required device memory is allocated for performing the sorting, scan, and reduction.

In the serial solution, a STL (*C*++ Standard Template Library) container, vector<int>, is adopted to allocate an array to dynamically store the indices of neighboring nodes for each vertex. The size of the array can be dynamically determined without redundant space. Similar, another array of integers is needed to hold the indices of neighboring elements. Moreover, the number of neighboring nodes and elements are directly the size of the dynamic arrays, which can be easily and automatically determined. Thus, there is no need to allocate other additional arrays.

In contrast, in our parallel solution, six additional arrays of integers are required. First, two arrays of integers need to be allocated to store the edges (i.e., pairs of integers). Second, another two arrays of integers are needed to hold the first indices and numbers of neighboring nodes. And third, to perform the segmented parallel reduction and scan, another two temporary arrays are also required.

Due to the required additional arrays of integers, our parallel solution cannot be applied to quite large size of meshes since the device memory (the global memory) of most current GPUs is quite limited. Thus, future work may focus on redesigning the parallel process of finding nodal neighbors to reduce the device memory.

## Conclusions and outlook

In this paper, we have presented a parallel solution to finding the neighboring nodes and elements for each vertex in an arbitrary mesh by exploiting the GPU. Our solution is a topology-based method, and is heavily dependent on the use of parallel sorting, scan, and reduction. We have compared our parallel solution to the corresponding serial solution to evaluate its performance. We have found that: our parallel solution is approximately 55 and 90 times faster than the corresponding serial solution when finding the neighboring nodes and elements, respectively. Our solution is efficient, simple and easy to implement, and can be applied for arbitrary meshes. However, our parallel solution requires the allocation of large device memory; and thus future work is planned to be carried out to address this problem.

## Additional information

**Introduction**

Mesh generation plays an important role in geometric modeling, computer graphics, and numerical simulations. After generating various types of meshes, typically mesh editing is intentionally performed to modify or improve the generated meshes to meet desired requirements. In mesh editing such as Boolean operations [8] or mesh optimization [9], the local mesh connectivity especially the adjacent/neighboring nodes and elements for each node or element is frequently needed to reduce the computational cost of local search.

The finding of one-ring nodal neighbors in arbitrary valid mesh is computationally straightforward, and can be completely carried out based on the connectivity/topology of meshes. The simplest method is to loop over all elements in a mesh to identify: (1) which pair of nodes is connected by an edge and (2) which nodes are contained in an element [4,9,10]. This is because that: (1) any pair of nodes connected by an edge is the one-ring neighboring node for each other; and (2) any element is directly the one-ring neighboring element for those nodes it contains.

Another simple method for finding the one-ring neighboring nodes for each vertex in a polygonal mesh was introduced by Dahal and Newman [11]. They first determined the boundary vertices by finding the opposite edges for each vertex and then forming a closed polygon using those opposite edges. They adopted the vertices of the closed polygon as the one-ring neighbors for each vertex.

Both of the above neighbors-finding methods are easy to implement in the serial programming pattern. However, due to the fact that it needs to loop over all the elements of a mesh in sequential, the computational cost is in general too high for large size of meshes; and this will reduce the computational efficiency of the entire mesh editing procedure.

An effective strategy to improve the efficiency of the neighbors-finding procedure is to parallelize it on various parallel computing architectures such as multi-core CPUs or many-core GPUs.

However, when finding nodal neighbors in parallel, there exists the race condition. The race condition issue appears when two different parallel threads may need to be written in the same memory position [3]. When looping over all the elements in a mesh to find the nodal neighbors, two neighboring nodes of the same vertex may be found concurrently within two parallel threads; and the indices of the two neighboring nodes may need to be written in the same memory position for storing. In this case, race condition arises.

Currently, the most commonly used method to address the above problem is to color the mesh first and then looping over those elements with the same color simultaneously to find neighbors [3,4,5,6,7]. This coloring-based method is very effective and efficient for large meshes, and is quite suitable to be applied in parallel pattern. The only minor shortcoming is that: it needs additional computational cost to color the mesh into several groups of elements.

In this paper, without the use of mesh-coloring, we specifically design and develop a parallel solution to finding nodal neighbors by utilizing the power of modern GPUs. Our solution is efficient, simple and easy to implement, which heavily depended on the use of parallel primitives such as sorting, scan, and reduction. In addition, in our solution there is no need to adopt complex mesh data structures; and only arrays of integers are required. To evaluate the performance of our parallel solution, we compare it to the corresponding serial solution in six experiments.

## Declaration of Competing Interest

The authors declare that they have no known competing financial interests or personal relationships that could have appeared to influence the work reported in this paper.
